# Endourologic Diagnosis and Robotic Treatment of a Giant Fibroepithelial Polyp of the Ureter

**DOI:** 10.1089/cren.2016.0100

**Published:** 2016-10-01

**Authors:** Francesco Cattaneo, Fabio Zattoni, Luca Meggiato, Claudio Valotto, Fabrizio Dal Moro, Marina Paola Gardiman, Paolo Beltrami, Filiberto Zattoni

**Affiliations:** ^1^ Department of Surgery, Oncology, and Gastroenterology, Urology Clinic, University of Padua, Padua, Italy.; ^2^ PhD Course in Clinical and Experimental Oncology and Immunology, Department of Surgery, Oncology, and Gastroenterology, University of Padua, Padua, Italy.; ^3^ Department of Medicine, Surgical Pathology and Cytopathology Unit, University of Padova, Padova, Italy.

**Keywords:** fibroepithelial polyp, endourology, robot-assisted laparoscopy, ureteropelvic junction obstruction

## Abstract

***Background:*** Fibroepithelial polyps (FEPs) are a rare cause of ureteropelvic junction (UPJ) obstruction. Radiologists and urologists are not always confident with this disease because of its rarity, complex diagnosis, and heterogeneity of the available treatment options.

***Case Presentation:*** We present the endourologic diagnosis and the robotic management of a ureteral polyp close to the left UPJ. A 16-year-old woman with a 12 years history of left lumbar pain was referred to our Center. A computed tomography scan detected a left hydronephrosis with no signs of obstructions at MAG-3 scintigraphy. The endourologic evaluation revealed a giant FEP of the left ureter, which was removed surgically with a videolaparoscopic robot-assisted approach.

***Conclusion:*** Considering that conventional radiologic imaging techniques can hardly detect a ureteral FEP, an endourologic study of the urinary tract is mandatory to directly observe the polyp. The mini-invasive treatment of ureteral FEPs is feasible and safe, and should be considered as first option in young patients.

## Introduction and Background

Ureteropelvic junction obstruction (UPJO) is defined as impaired urine flow from the pelvis into the proximal ureter because of congenital ureteral malformation or crossing vessels. Possible consequences of UPJO are the dilation of the collecting system and the risk of kidney failure. Indications for surgical correction are decreased renal function, stone development, urinary infections, or other clinical associated symptoms (e.g., abdominal pain, hematuria, dysuria, and flank discomfort).^1^

Fibroepithelial polyps (FEPs) are a relatively rare cause of UPJO in the pediatric population, with an incidence reported of 0.5%. FEPs are more common in boys than in girls and they usually develop in the left side (up to 70%).^2^

FEPs are benign mucosal projections of fibrous stroma lined by surface epithelium. They may involve the renal pelvis, ureter, bladder, or urethra. Different phenotypes of FEPs have been described: some are long, cylindrical masses, whereas others are shorter, wider, and more likely to cause urinary obstruction.^1,2^

Although the exact etiology of FEPs is unknown, chronic irritation or infections, developmental or allergic factors, trauma, or congenital causes could be implicated.

These entities may mimic the symptoms of intrinsic UPJO and are often undiagnosed until the time of pyeloplasty. Moreover, urothelial cancer should always be suspected until a definitive diagnosis is obtained.^2^

UPJO caused by FEPs requires surgical treatment and is even considered the risk of malignity. Nowadays, the use of minimally invasive techniques is widespread and currently robotic, laparoscopy, transurethral ureteroscopy, and percutaneous access are replacing the more conventional open surgery.^2^

## Presentation of Case

A 16-year-old woman was admitted to our tertiary referral Center for a 12-year history of occasional, slight lumbar pain involving the left upper side of the abdomen. No hematuria, nausea, vomiting, or fever was documented. A urinalysis showed calcium oxalate crystals in the urinary sediment suspicious for kidney stones.

An abdominal ultrasonography (US) showed a left hydroureteronephrosis in the proximal ureter with initial thinning of renal parenchyma; neither stones nor intraluminal ureter lesions were recorded.

The next contrast computed tomography (CT) scan of the abdomen and pelvis confirmed the left hydronephrosis (renal pelvis anteroposterior diameter 35 mm). The left proximal ureter was also dilated up to 8 mm, without opacification on the excretory phase CT images. However, an MAG-3 renal scintigraphy showed a total left kidney function of 46%, with late and incomplete drainage after furosemide injection.

**FIG. 1.**
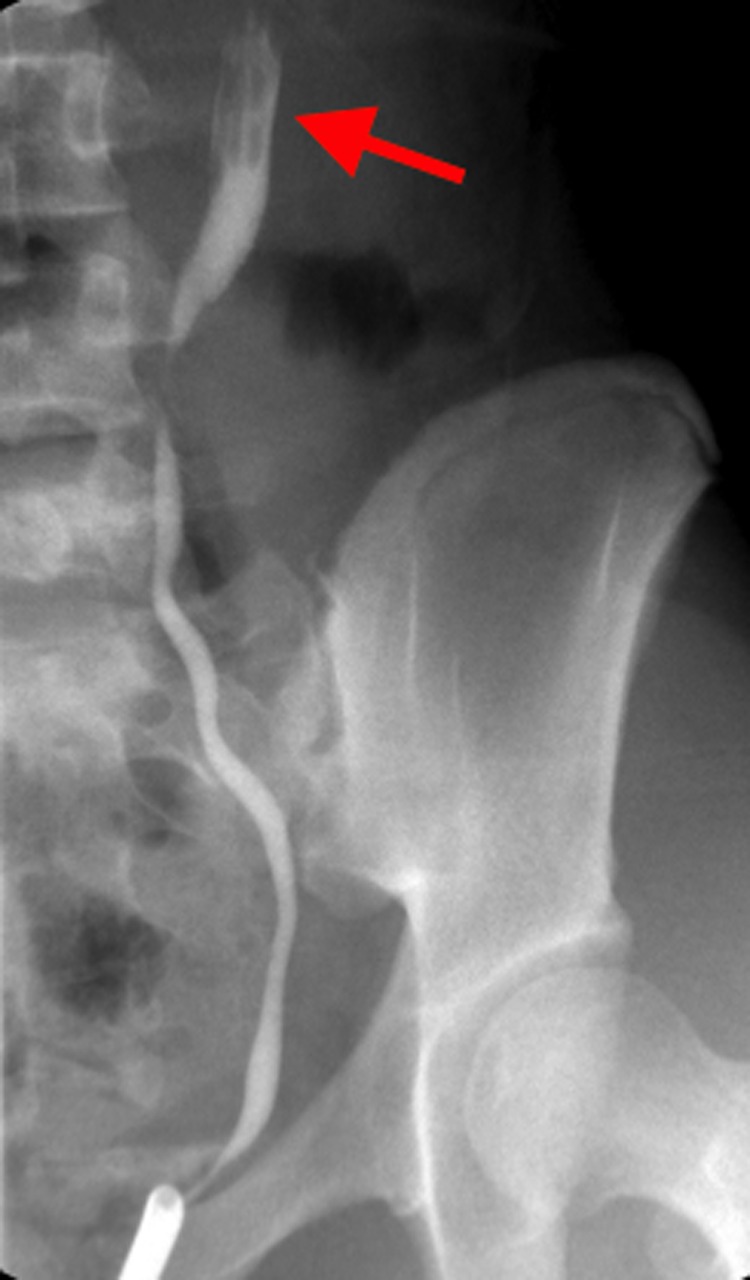
Retrograde pyelography reveals a filling defect (*arrow*) at the level of the ureteropelvic junction (UPJ).

**FIG. 2.**
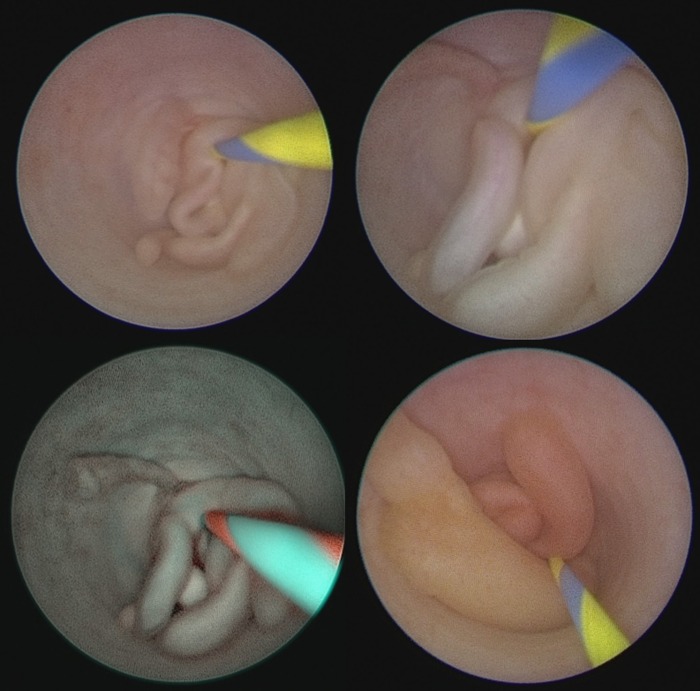
The endoscopic appearance of the fibroepithelial polyp at the UPJ with white light and in spectra modality (SPIES).

Before the robotic correction of suspected UPJO, we usually place a Double-J ureteral stent; an intraoperative retrograde pyelogram showed a filling defect in the left ureteropelvic junction (UPJ) (Fig. 1). The clinical diagnosis of FEP in the UPJ was finally achieved through a ureterorenoscopy (Fig. 2).

**FIG. 3.**
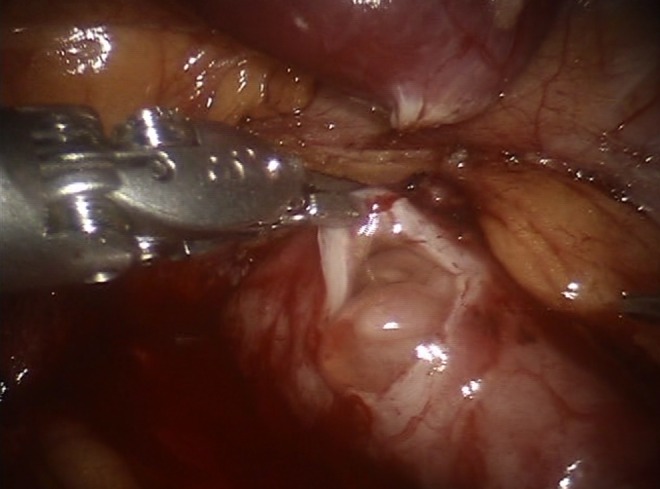
Intraoperative view of ureteral polyp protruding outside from the incision of UPJ.

The robotic surgical approach was performed immediately after the ureterorenoscopy; it was transperitoneal, placing the same surgical ports of robot-assisted laparoscopic pyeloplasty, with the patient positioned in the right lateral decubitus 60° rotated upwards. An intraperitoneal access was performed through open surgery nearly 1 cm lateral to the umbilicus. A 12 mm robotic camera trocar was inserted through an access track, and pneumoperitoneum was achieved with an insufflation pressure of 13 mm Hg CO_2_. The two operative robotic ports were placed, respectively, one between the anterior superior iliac spine and the umbilicus and the other on the pararectal line 1 cm beyond the costal arch. The 5 mm assistant port was positioned at the midpoint between the umbilicus and the xiphoid process. The white line of Toldt was cut and the left colon was medialized to expose Gerota's fascia, which was incised isolating the proximal ureter and the dilated renal pelvis. A transverse distal renal pelvis incision revealed an irregularly shaped 25 mm polyp, circumferentially involving the ureteral lumen (Fig. 3). The polyp appeared as a multiple finger-like projection arising from a large base. Excision of the mass was performed, including the regions of pelvis and ureter close to the mass. The ureter was then spatulated longitudinally and pyeloplasty was performed according to the Anderson–Hynes technique. The plasty was performed with an interrupted 5-0 Vicryl suture, on the Double-J catheter. At the end of the procedure, once complete hemostasis was achieved, a suction drain was left in place.

The total operative time was 92 minutes, with an estimated operative blood loss of 50 mL. The drain was removed on the second postoperative day and the patient was discharged on the fifth postoperative day. The ureteral stent was removed after 4 weeks. Neither immediate nor late postoperative complications occurred.

**FIG. 4.**
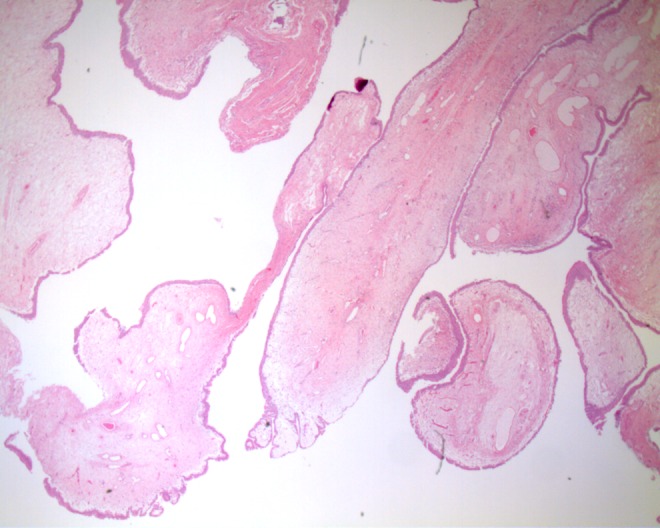
High power view of section from the tissue shows a polypoid structure lined by benign transitional epithelium.

Pathologic examination confirmed a polypoid FEP lined by benign transitional epithelium with marked edema of the underlying stroma (Fig. 4).

After a 6-month follow-up, the patient showed complete recovery with normal US and MAG-3 renal scintigraphy.

## Discussion and Literature Review

FEPs are benign ureteral lesions of mesodermal origin. They may arise in any location from renal pelvis to the urethra with decreasing frequency.^1^

The etiology of ureteral FEPs is still debated. Various hypotheses have been proposed, including congenital condition, chronic irritation, stones, infections, obstruction, trauma, and hormonal or developmental disturbances.^1^

Previously, ureteral polyps causing UPJO have been reported in as low as 0.5% of the pyeloplasties performed in children.^2^ However, in recent studies, incidence of FEPs was reported to be around 5.2% in the general population. As Li et al. mentioned in their report, it is unclear whether this represents an increasing incidence of ureteral FEPs, improved detection, or publication bias.^2^ Interestingly, FEPs seem to occur more frequently in males (92.0%) with a predilection for the left ureter (67.0%); bilateral cases are reported as well.^1,2^

If large enough, FEPs can cause urinary obstruction with ipsilateral flank pain as the most common presenting symptom (76%). Other less frequent clinical presentations include microscopic hematuria (19.7%), pyuria, urinary tract infections, neonatal flank mass, and hydronephrosis. In rare cases, torsion of the polyp may cause severe ischemic pain.^2^

Common diagnostic tools include US, CT scan, magnetic resonance imaging (MRI), renal scintigraphy, and retrograde pyelogram. However, the sensitivity of US, urography, and MRI is reported to be low (49%).^2^ Possible causes of the low accuracy of imaging in the preoperative setting may be because of several reasons: small size of the polyp, very low incidence, and radiology inexperience. In our case, neither US nor CT scan was effective in detecting the FEP, whereas we were able to identify the filling defect through a retrograde pyelogram, according to the 80% sensitivity reported in recent studies.^2,3^

When a filling defect indicative of ureteral polyps is detected by means of retrograde pyelogram, ureterorenoscopy is mandatory for both diagnostic and therapeutic purposes; in fact, the treatment strategy is driven by the endoscopic appearance of the ureteral polyp.^3^

Although open surgery was the only option for the treatment of ureteral FEPs, recently minimal invasive approaches have been recommended in children and adults. Pedunculated and solitary polyps can be effectively treated endoscopically with percutaneous and/or ureteroscopic approaches using a Holmium:YAG laser. Laparoscopy should be the treatment of choice for large, multilobulated or broad-based FEPs. Laparoscopic and laparoscopic robot-assisted surgeries are minimal invasive techniques that can provide satisfactory outcomes in patients with polyps localized in the ureter or in the UPJ.^4^

## Conclusion

In this report, we present the endoscopic and laparoscopic appearance of an UPJ FEP. Since the preoperative imaging techniques are poorly diagnostic, an endourologic study of the urinary tract is the only way to directly observe the FEP.

As far as the treatment is concerned, robotic pyeloplasty is safe and effective in patients with UPJO because of benign FEPs. In addition, the use of the robot-assisted laparoscopy allows a clear intraoperative vision, a minimally invasive surgical approach (small incisions, minimal blood loss, faster recovery, and optimal esthetic results), and a complete resection of FEPs. Even though this is a single case experience, robot-assisted polypectomy seems to be a valid surgical option for treatment of FEPs.

## Abbreviations Used

CT: computed tomography
FEP: fibroepithelial polyp
MRI: magnetic resonance imaging
UPJ: ureteropelvic junction
UPJO: ureteropelvic junction obstruction
US: ultrasonography
